# Coronectomy of impacted mandibular third molars: A meta-analysis and systematic review of the literature

**DOI:** 10.4317/medoral.21074

**Published:** 2016-03-31

**Authors:** Juan Cervera-Espert*, Sara Pérez-Martínez*, Juan Cervera-Ballester, David Penarrocha-Oltra, Miguel Penarrocha-Diago

**Affiliations:** 1 Resident of the Master in Oral Surgery and Implant Dentistry, Stomatology Department, Faculty of Medicine and Dentistry, University of Valencia, Spain; 2 Master in Oral Surgery and Implant Dentistry, Faculty of Medicine and Dentistry, University of Valencia, Spain; 3 Collaborating Professor of the Master in Oral Surgery and Implant Dentistry, Stomatology Department, Faculty of Medicine and Dentistry, University of Valencia, Spain; 4 Chairman of Oral Surgery, Stomatology Department, Faculty of Medicine and Dentistry, University of Valencia, Spain

## Abstract

**Background:**

Coronectomy is an alternative to complete removal of an impacted mandibular third molar. Most authors have recommended coronectomy to prevent damage to the inferior alveolar nerve during surgical extraction of lower third molars. The present study offers a systematic review and metaanalysis of the coronectomy technique.

**Material and Methods:**

A systematic review and meta-analysis was performed based on a PubMed and Cochrane databases search for articles published from 2014 and involving coronectomy of mandibular third molars located near the inferior alveolar nerve canal, with a minimum of 10 cases and a minimum follow-up period of 6 months. After application of the inclusion and exclusion criteria, a total of 12 articles were included in the study.

**Results and Discussion:**

Coronectomy results in significantly lesser loss of sensitivity of the inferior alveolar nerve and prevents the occurrence of dry socket. No statistically significant differences were observed in the incidence of pain and infection between coronectomy and complete surgical extraction. After coronectomy, the remaining tooth fragment migrates an average of 2 mm within two years.

**Conclusions:**

Coronectomy is indicated when the mandibular third molar is in contact with the inferior alveolar nerve and complete removal of the tooth may cause nerve damage.

**Key words:**Coronectomy, included third molar, inferior alveolar nerve injury.

## Introduction

Coronectomy was introduced by Knutsson *et al.* (1) as an alternative to complete removal of an impacted mandibular third molar. The technique removes only the crown, leaving the root in the socket, and preventing direct or indirect damage to the inferior alveolar nerve (IAN). Injury to the IAN is a rare but serious complication of mandibular third molar extraction, with an incidence ranging from 0.5% to 8% (2-4) . Injury proves permanent in 1% of the cases (4,5).

Computed tomography (CT) or cone beam computed tomography (CBCT) can determine the exact relationship between the inferior alveolar nerve and the third molar (6). Computed tomography shows a direct association between inferior alveolar nerve injury and the absence of cortical bone between the roots and the inferior alveolar canal (7,8). Many authors have recommended coronectomy to prevent damage to the inferior alveolar nerve during the surgical extraction of third molars that are in contact with the nerve (8-10). The realization of a meta-analysis for synthesizing all the data published in the literature is required.

- Aim

The present study offers a systematic review and statistical data for a meta-analysis of coronectomy of the mandibular third molar as a technique for avoiding permanent damage to the inferior alveolar nerve and the appearance of other complications.

## Material and Methods

A PubMed and Cochrane Library search following the PRISMA guidelines was made for articles on coronectomy in impacted mandibular third molars. Studies published in English or Spanish to December 2014 were reviewed. The keywords used were a combination of “coronectomy AND third molar.”

- Focus question

The focus question was established according to the PICO (population, intervention, comparison, outcome) format: In patients with third molar in contact with the IAN, coronectomy prevents further injury IAN and the appearance of other complications compared to the complete surgical removal?

• P (population): patients with third molar in contact with the IAN.

• I (intervention): third molar surgery.

• C (comparison): coronectomy vs complete removal.

• O (outcome): prevents further injury IAN and the appearance of other surgical complications.

- Inclusion criteria:

Clinical studies in humans comparing the effects of coronectomy versus complete surgical removal of third molars in contact with the mandibular nerve were reviewed. The type of included articles were randomized clinical trials (RCT, controled clinical trials (CCT) and prospective cohort studies (PCS), prospective (PS) and retrospective (RS) studies with or without control group. The included studies must have a sample with a minimum of 10 coronectomy procedures performed with a minimum follow-up period of 6 months, and with the full text available in English or Spanish

- Exclusion criteria

Case reports, in vitro studies, comments to authors and literature reviews were excluded.

- Article selection

**Figure 1:**
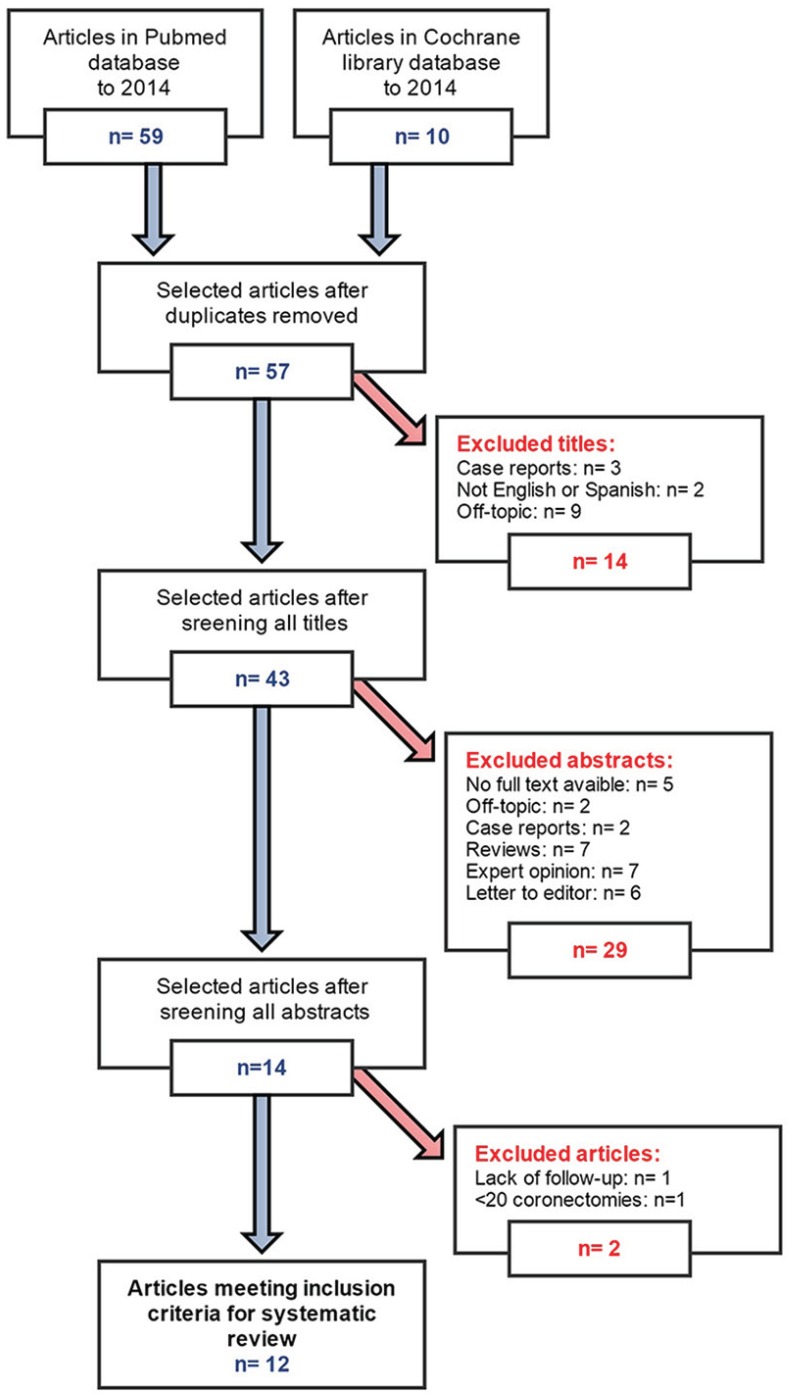
Flow chart for the systematic review.

After the initial search, a total of 57 non repeated articles were obtained with the keywords “coronectomy and lower third molar.” Forty-five studies were excluded systematically after scenning title, abstracts and full articles consecutively (Fig. 1).

The reasons for exclusion were as follows: 5 cases reports, 2 studies in a language other than English or Spanish, 5 publications lacking the full text article, 7 expert opinions, 6 letters to editor, 11 studies unrelated to the subject, 7 literature reviews, 1 study without the required minimum of ten coronectomy procedures and 1 study without the required minimum follow-up period of six months.

Twelve studies were thus finally included, with evaluation of the following parameters in all of them (including standard deviation): number of patients, number of extractions and coronectomy procedures, follow-up period; and the most frequently reported complications in the included articles: loss of sensitivity of the inferior alveolar nerve, onset of pain, infection or dry socket, and migration of the roots after coronectomy ( Table 1).

**Table 1:**
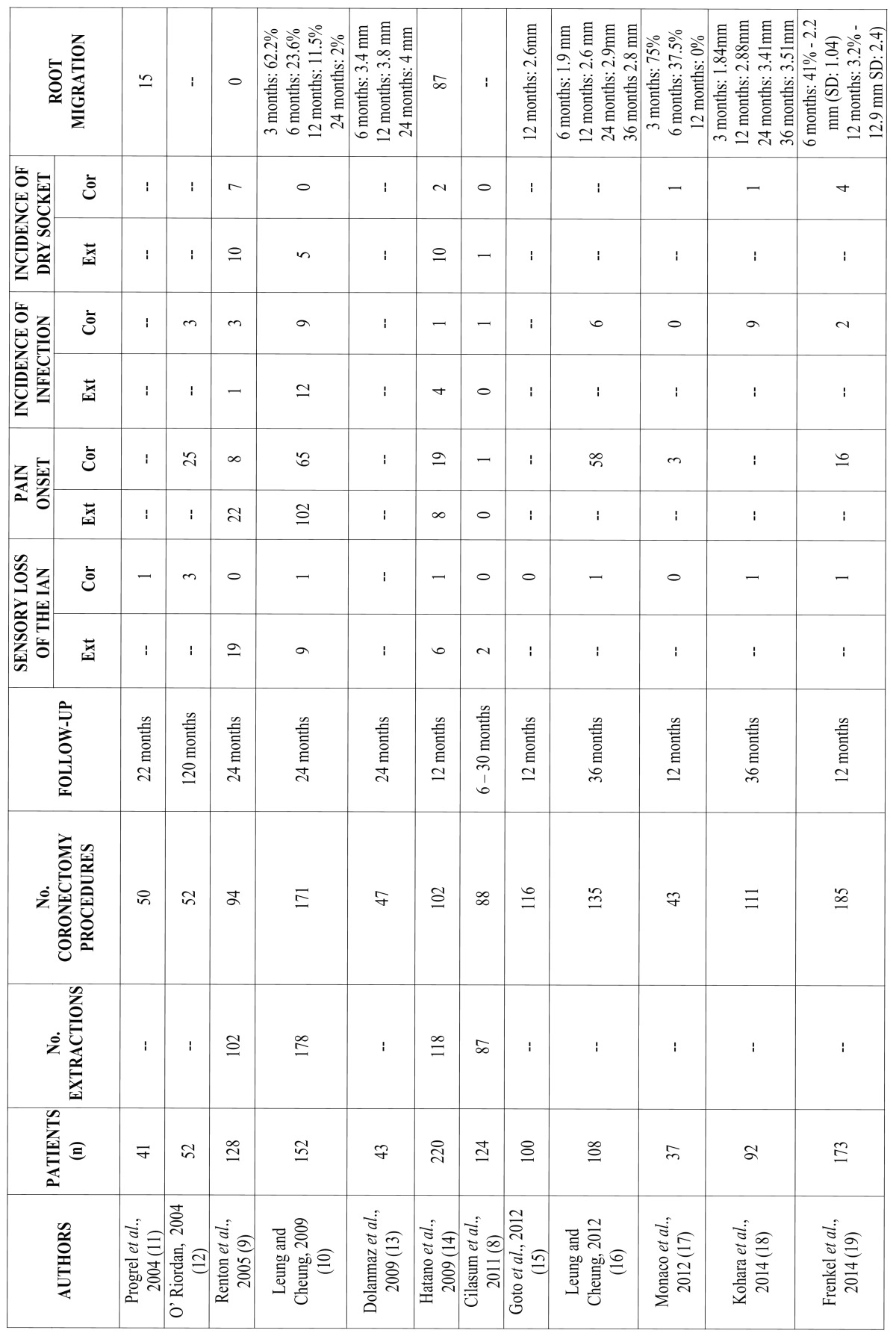
Studies included in the systematic review.

Articles were obtained from the following journals: *“Journal of Oral and Maxillofacial Surgery”, “Oral Surgery, Oral Medicine, Oral Pathology, Oral Radiology & Endodontics”, “British Journal of Oral and Maxillofacial Surgery”, “Journal of the American Dental Association”, and “International Journal of Oral and Maxillofacial Surgery”*. Assessment of study quality and risk of bias. 

All eligible studies were assessed for methodological quality by two independent reviewers. The overall risk of bias was analyzed for such randomized clinical trials or quasi-experimental trials ( Table 2) that are considered moderate risk of bias is determined whether a domain and high bias if it is determined in more than one domain. For cohort studies, prospective or retrospective case series and other observational studies ( Table 3) they were rated as high risk of bias if they have 1 point or less on the Newcastle-Ottawa Quality Assessment Scale (NOQAS). Studies were included if quality analysis was 2 or more.

**Table 2:**
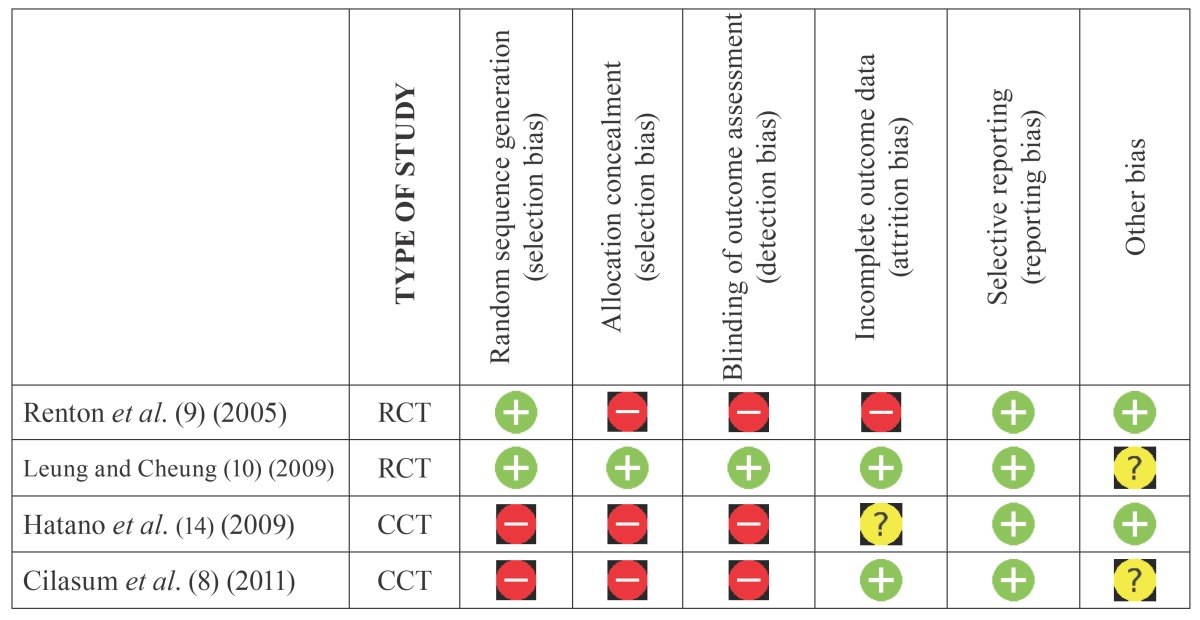
Risk of bias assessment of the RCTs and quasi-experimental studies with the recommended approach of the Cochrane colaboration.

**Table 3:**
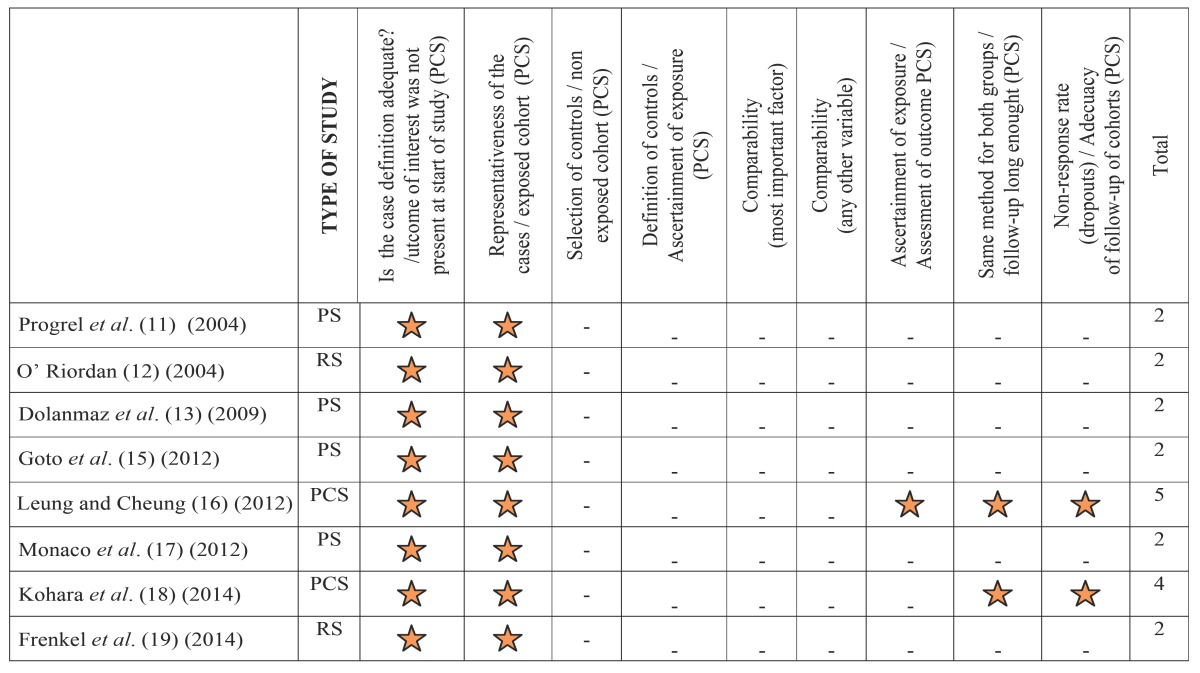
NOQAS to risk of bias assessment for observational studies.

- Metaanalysis and statistical analysis

A metaanalysis was performed to obtain the measure of an overall effect (odds ratio, OR). The overall effect were only mesured in randomized clinical trials (RCT, controled clinical trials (CCT) and prospective cohort studies (PCS). Also, a metaregression analysis was performed to assess the effect of the technique upon the probability of complications about all the included articles: loss of sensitivity of the inferior alveolar nerve, onset of pain, infection or dry socket, and migration of the roots after coronectomy.

The study of heterogeneity was made by I2 statistical calculation. For the evaluation of bias in studies, Funnel plots and Egger’s test were used. A *p* value of the Q statistic that was less than 0.10 was considered significant. I2 values of 25%, 50%, and 75% correspond to cutoff points for low, moderate, and high degrees of heterogeneity. The metaanalysis was conducted using the R 3.0.2 application. The level of significance used in the analysis was 5% (α=0.05).

## Results

- Loss of sensitivity of the inferior alveolar nerve:

In eleven of the included studies (8-10,11-13,14-19) the occurrence of sensitivity loss of the inferior alveolar nerve was evaluated after complete surgical removal of the lower third molar and after coronectomy ( Table 1). Of these studies, four (8-10,14) compared the loss of sensitivity between full extraction and coronectomy of the lower third molar, recording a more cases of sensitivity loss of the inferior alveolar nerve in complete extractions. Regarding the rest of the articles, Goto *et al.* (15) and Monaco *et al.* (17) recorded no cases of sensitivity loss of the inferior alveolar nerve with the coronectomy technique. Two studies (10,11) reported transient paresthesia of the lingual nerve. Kohara *et al.* (18) reported only a one case of paresthesia (0.9%); and O’Riordan *et al.* (12) documented three temporary sensitivity losses in 52 coronectomy procedures (5.5%) - only one of which proved permanent (1.8%). Frenkel *et al.* (19) in turn reported only one case of temporary inferior alveolar nerve paresthesia in the first month after coronectomy.

**Figure 2:**
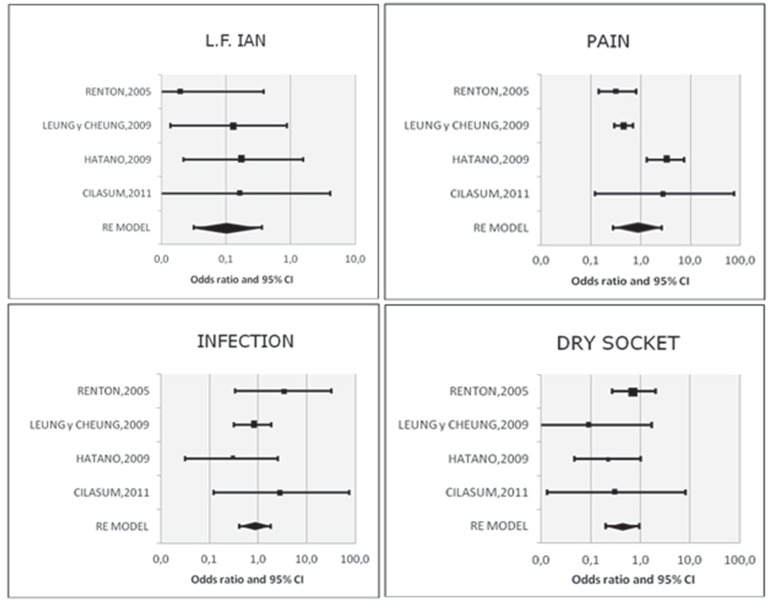
Forest plot for the incidence of the different complications (OR).

On selecting for the metaanalysis the four articles that compared coronectomy versus complete extraction (8-10,14), the Forest plot yielded OR=0.11 (Fig. 2) with a 95% confidence interval (95% CI) of 0.03-0.36 (*p*<0.001). In this regard, the coronectomy technique reduced the risk of sensitivity loss by 89% compared to extractions. The studies were homogeneous (I2=0.0% and Egger’s test: *p*=0.652).

**Figure 3:**
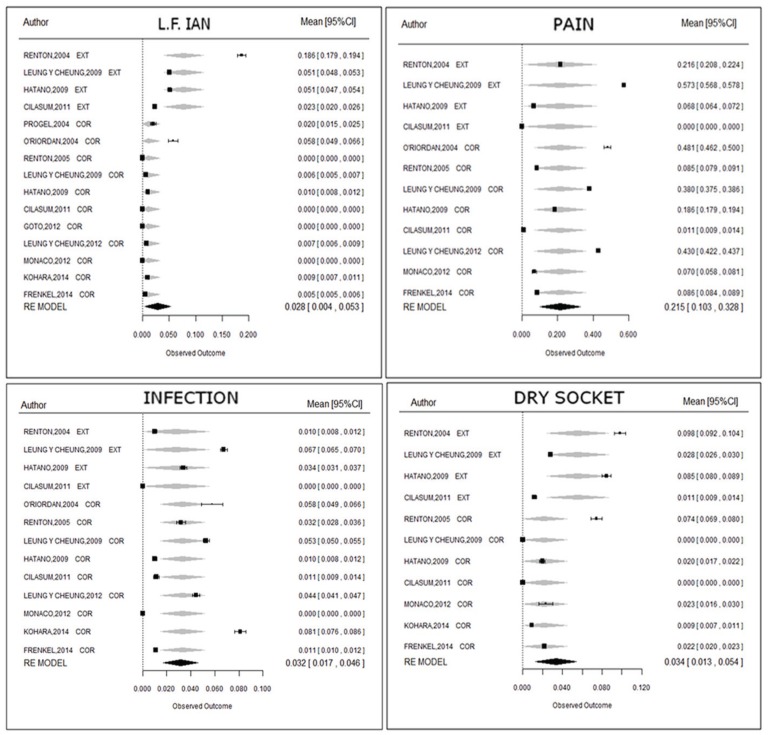
Forest plot for the incidence of the different complications (metaregression analysis).

On separately considering the two techniques, a total of 15 publications were seen to report information on sensitivity loss of the inferior alveolar nerve (4 studies on complete third molar removal and 11 coronectomy studies), representing a total of 1632 operations. Metaregression analysis of these data (Fig. 3) revealed a significant effect of the technique employed upon the probability of sensitivity loss (*p*=0.003, Q Omnibus test).

- Pain:

Eight of the included studies evaluated the onset of pain (8-10,12,14,16,17,19). Renton *et al.* (9) and Leung and Cheung (10) registered more patients with pain after complete removal of lower third molars than after coronectomy, though the difference was not statistically significant. In contrast to the above authors, Hatano *et al.* (14) and Cilasum *et al.* (8) reported increased pain in patients subjected to coronectomy versus complete extraction. Other authors only registered pain in relation to coronectomy (10,12,17), with pain incidences of 48% (12), 42.9% (16), 7% (17) and 9.2% (19) during the first month after surgery. Lastly, Monaco *et al.* (17), in a group of 37 patients, reported pain in three cases in the first week. In one case second surgery was needed to remove root fragments 10 months after coronectomy, due to apical infection.

On selecting for the metaanalysis the four articles that compared coronectomy versus complete extraction (8-10,14), the Forest plot yielded OR=0.87 (Fig. 2), with 95%CI 0.28-2.66 (nonsignificant; *p*=0.803). There is not enough statistical evidence to affirm that coronectomy reduces the level of pain. The studies were not particularly homogeneous about pain, though estimating I2=83.3%, the studies pointed in one direction or other. Egger’s test confirmed the absence of bias (*p*=0.414).

On separately considering the two techniques, a total of 12 articles were seen to offer information on pain, comprising a total of 1355 interventions. Metaregression analysis of these data (Fig. 3) showed that there is not enough statistical evidence of a significant effect of the technique upon the probability of pain (*p*=0.987, Q Omnibus test).

Infection:

The occurrence of infection with the presence of pus after treatment was assessed in nine of the studies (8-10,12,14,16-19). The articles by Leung and Cheung (10) and Hatano *et al.* (14) showed more infections with pus in patients subjected to complete surgical removal of impacted thirds molars in contact with the inferior alveolar nerve versus those subjected to coronectomy. In contrast, Renton *et al.* (9) and Cilasum *et al.* (8) recorded a larger number of patients with infection after coronectomy than after complete extraction. O’Riordan (12) and Leung and Cheung (16) registered only the infections produced after coronectomy, with incidences of 5.7% and 4.4%, respectively. Monaco *et al.* (17) recorded no infections associated to coronectomy in their study. Kohara *et al.* (18) described 7 incomplete wound exposures (6.3%) and two root exposures with infection (1.8%) that had to be removed. Lastly, Frenkel *et al.* (19) registered two infections with pus (1.1%) in the first month after 185 coronectomy procedures.

On selecting for the metaanalysis the four articles that compared coronectomy versus complete extraction (8-10,14), the Forest plot yielded OR=0.87 (Fig. 2) with 95% CI 0.41-1.84, (nonsignificant; *p*=0.707). There is not enough statistical evidence to affirm that coronectomy reduces the incidence of infection. The studies were homogeneous (I2=0.0% and Egger’s test: *p*=0.539).

On separately considering the two techniques, a total of 13 articles were seen to offer information on infection, comprising a total of 1466 interventions. Metaregression analysis of these data (Fig. 3) showed that there is not enough statistical evidence of a significant effect of the technique upon the probability of infection (*p*=0.747, Q Omnibus test).

- Dry socket:

In the literature included in our systematic review, only seven authors (8-10,14,17-19) recorded the occurrence of dry socket, which proved more frequent in patients subjected to complete surgical extraction than in those subjected to coronectomy. Monaco *et al.* (17) documented one dry socket in a series of 43 coronectomy procedures (2.43%), while Kohara *et al.* (18) only reported one dry socket in 116 procedures (0.86%), and Frenkel *et al.* (19) documented three cases of incomplete socket healing in 185 procedures.

On selecting for the metaanalysis the four articles that compared coronectomy versus complete extraction (8-10,14), the Forest plot yielded OR=0.44 (Fig. 2) with 95%CI 0.20 -0.96 (*p*=0.040). The coronectomy technique was seen to reduce the risk of dry socket compared with complete third molar removal. The studies were homogeneous (I2=0.0% and Egger’s test: *p*=0.192).

On separately considering the two techniques, a total of 11 articles were seen to offer information on dry socket, comprising a total of 1279 interventions. Metaregression analysis of these data (Fig. 3) showed that there is not enough statistical evidence of a significant effect of the technique upon the probability of dry socket (*p*=0.086, Q Omnibus test) - though the result came close to significance.

- Root migration:

Eleven of the included articles (8-10,11,13-19) studied root migration after coronectomy. Progel *et al.* (11) compared the radiographs at the time of coronectomy versus 6 months after the procedure, recording fragment root migrations of 2-3 mm from the initial position in 30% of the cases. Leung and Cheung (10) and Monaco *et al.* (17) registered the highest percentage (62.2% and 75%, respectively) of third molar root fragment migration in the third month after coronectomy (with an average distance of 1.90 mm and 1.60 mm, respectively). Moreover, Dolanmaz *et al.* (13) and Leung and Cheung (16) found maximum migration of the root fragments to occur two years after coronectomy (with an average distance of 4 mm and 2.90 mm, respectively). After the second year the degree of migration was greatly reduced, showing no statistically significant correlation to either patient age or gender. Kohara *et al.* (18) recorded greater root migration in the first two years (average 1.84 mm in three months, 2.88 mm in one year, and 3.41 mm and 3.51 mm after two years). From the second year after surgery, 82.2% of the roots did not move. Regarding gender, Goto *et al.* (15) and Leung and Cheung (16) reported significantly increased root migration in female patients. Frenkel *et al.* (19) in turn reported significantly greater migration in younger patients.

The studies did not provide homogeneous data for conducting a metaanalysis of root migration.

Discussion

In performing the meta-analysis, they have only won four articles to determine the odds ratio, two RCTs (9,10) and two CCTs (8,14), new RCTs with low risk of bias are needed in order to reinforce the results of this work. Besides, were found many studies of case series without control group (11-13,15-19), these studies have shown high levels of bias for meta-regression.

Results in the meta-analysis about the loss of feeling the inferior alveolar nerve, both the OR value as the result of metaregression shown statistically significantly less risk. The results about pain and infection no shown statistically significant differences between the performance of coronectomies and the complete extraction of the included third molar. About the incidence of dry socket the OR value shown a statistically significantly less risk in the coronectomies, the results of the metaregression only shown a trend to statistical significance but the metaregresion it includes studies with a higher level of bias regarding the OR.

The inferior alveolar nerve injury is rare, but is a well known complication during conventional extraction of mandibular third molar to be in intimate contact. Risk factors for nerve injury are known to include radiographic proximity, the surgeon’s experience, surgical procedures, the patient’s age, and preexisting disease (20). This technique is performed to remove only the crown, leaving the root in the socket. The traditional method used for planning the extraction of third molar is a panoramic radiography, the CBCT being the most accurate method to determine the exact relationship between the inferior alveolar nerve and third molars root. Using the CBCT as diagnostic element with coronectomies performing can avoid the inferior alveolar nerve injury in the included third molars in contact with the inferior alveolar nerve.

Other reviews in the literature about coronectomies were performed, but there is only one systematic review (21) and one meta-analysis (22). Suggest that coronectomy can protect inferior alveolar nerves in the extraction of third molars with high risk of nerve injury as compared with total removal, and that the risk ratios of post-operative infections were similar between the two surgical modalities. Although root migration rate was high (13.2%-85.29%), the migration distances were short (3.06 ± 1.67 mm), and the directions were away from the nerves. Moreover, the rates of re-operation and root exposure were low (21).

In a bibliographical reviews, Moreno-Vicente *et al.* (23) conclude that the coronectomy is an adequate preventative technique in IAN protection. It is shown as an alternative to the conventional extraction of third molars in which there is a high risk of injury to the inferior alveolar nerve. Auyong and Le (24) refers a lower incidence of neurosensory damage associated with dentoalveolar surgery too. Furthermore, this technique has fewer complications compared to complete removal (25).

This technique only should be applied in specific cases, coronectomy is particularly appropriate for patients older than 25 years, and who report low tolerance for the possibility of posttreatment neurosensory deficit at the consultation (26). The issue of iatrogenic inferior alveolar nerve damage during the removal of lower third molars continues to be a clinical and medico-legal problem is  as a valid treatment option in high risk cases (27).

A point of discussion is whether it is necessary then reoperation to remove the root fragments, or whether to perform a root canal simultaneously to coronectomy. Sencimen *et al.* (25) conclude that endodontic treatment does not affect the success of coronectomy method according their results.

## Conclusions

Coronectomy is indicated when the third mandibular molar is in contact with the inferior alveolar nerve, and where complete removal may cause injury to the nerve. Within its limitations the present systematic review and meta-analysis concluded that coronectomy results in a significantly lesser incidence of both sensitivity loss of the inferior alveolar nerve and dry socket. No statistically significant differences were observed in the incidence of pain and infection between coronectomy and complete molar removal.
